# Dynamic changes in the transcriptome of *Populus hopeiensis* in response to abscisic acid

**DOI:** 10.1038/srep42708

**Published:** 2017-02-15

**Authors:** Zhong Chen, Lexiang Ji, Jia Wang, Jinpu Jin, Xiaoyu Yang, Pian Rao, Kai Gao, Weihua Liao, Meixia Ye, Xinmin An

**Affiliations:** 1 National Engineering Laboratory for Tree Breeding, Key Laboratory of Genetics and Breeding in Forest Trees and Ornamental Plants of the Ministry of Education, The Tree and Ornamental Plant Breeding and Biotechnology Laboratory, College of Biological Sciences and Biotechnology, Beijing Forestry University, Beijing, 100083, P. R. China; 2 Institute of Bioinformatics, University of Georgia, Athens, GA 30602, USA; 3 State Key Laboratory of Protein and Plant Gene Research, Center for Bioinformatics, College of Life Sciences, Peking University, Beijing, 100871, P. R. China

## Abstract

Abscisic acid (ABA) plays a fundamental role in plant response and adaptation to abiotic stresses, such as drought, high salinity and low temperature. *Populus hopeiensis* exhibits exceptional tolerance to water-deficit environments and is therefore an excellent choice for studying drought tolerance in trees. This study provides a global view of transcriptome dynamics in *P. hopeiensis* in response to exogenous ABA using Illumina RNA-sequencing. Endogenous ABA content increased and reached a peak at 8 h after ABA treatment and then significantly decreased at latter time points. Differential expression analysis and Gene ontology enrichment revealed that the number of transcripts exhibited significant increase during the first 8 hours after ABA treatment, which then significantly decreased at 12 and 24 h. Transcription factors (TFs) analysis showed that six different patterns were observed based on the expression of the six TFs families (AP2/ERF, NAC, MYB, MYB-related, bZIP and WRKY) and the majority of differentially expressed TFs increased rapidly after ABA treatment. This study provides a robust resource for investigating the functions of genes induced by ABA and will help to develop a better understanding of the molecular regulatory mechanism in response to drought in poplar.

*Populus* is well suited for plant genomic studies due to its high level of genetic diversity and relatively small genome size. It is also one of the most widely distributed and cultivated woody plants because of its rapid growth rate, ease of vegetative propagation, and high woody quality, which makes it an ideal material for timber production and other forestry products^1^,^2^,^3^. Additionally, with its potential to be a sustainable and renewable cellulose-based biofuel, *Populus* is also regarded as a future alternative to fossil fuels^4^. However, abiotic stresses, such as drought, high salinity, and low temperature can have a substantial negative impact on the growth and productivity of *Populus*^5^. *P. euphratica* Oliv. (Salicaceae) which has high tolerance to salt and drought stress is naturally distributed in the desert areas of western China, it plays an important role in maintaining local arid ecosystems^6^,^7^. Similarly, another indigenous poplar with outstanding drought tolerance, *P. hopeiensis* Hu et Chow, is primarily distributed throughout northern and northwestern China, exhibits greater drought- and cold-tolerance than other aspen species^8^. Therefore, *P. hopeiensis* is considered as an ideal for elucidating the response mechanism under drought stress in woody plants.

Abscisic acid^9^, one of the major plant hormones, plays a fundamental role in plant response and adaptation to abiotic stresses, such as drought, high salinity and low temperature^10^,^11^. A previous study reported that nearly 10% of all the protein-coding genes in *Arabidopsis thaliana* are regulated by ABA^12^, highlighting its critical role in stress response. The study of ABA-related transcriptional regulation in plants, however, has been primarily focused on *A. thaliana*^11^,^12^, and studies on other species, especially woody plants such as *Populus*, are relatively limited^13^.

Thus far in the genus *Populus*, only the genome of *P. trichocarpa* has been sequenced, assembled and annotated^1^. The absence of a genome sequence in *P. hopeiensis* hinders its use for exploring its properties (such as drought tolerance) at a whole genome-wide scale. Fortunately, the rapid development of RNA-seq technology in recent years has provided an opportunity to systematically investigate the transcriptome and genome of a wide variety of species, including *P. hopeiensis*^14^,^15^. In the present study, we utilized RNA-seq technology to systematically analyze changes in the transcriptome of *P. hopeiensis* in response to ABA. Deep sequencing of paired-end libraries derived from leaf samples was used to conduct a time course study of the response of *P. hopeiensis* after exogenous treatment with ABA. The objective of the study was to provide insight into ABA-dependent related regulatory networks that are associated with water-deficit response in *P. hopeiensis*, a drought tolerant tree species, and to provide direction for future research in this area.

## Results and Discussion

### Deep sequencing and assembling result

Paired-end RNA-seq was used on leaf samples from tissue cultured plants of *P. hopeiensis* to characterize transcriptomic changes in response to ABA treatment. Samples were collected prior to ABA treatment, designated as Time 0 (US) and at 1, 4, 8, 12, and 24 h (A1-A5) after treatment with 100 μM ABA. In total, 245 million raw reads were generated using an Illumina platform, ranging from 38 to 45 million reads per sample per sample time (Table 1). After filtering out low-quality reads using an NGS toolkit^16^, 89.23% of the total reads were maintained as high-quality pair-reads. Due to the lack of a reference genome for *P. hopeiensis*, a *de novo* assembly strategy was used to construct the transcriptome. This assembly was used to identify transcripts and to quantify their abundance. Eventually, 204,390 transcripts with length greater than 200 nucleotides (nt) were identified. The identified transcripts had an average length of 1,120 nt and an N50 value of 1,873 nt. There were 83,402 (40.81%) transcripts with a length greater than 1,000 nt (Figure S1). To further estimate the quality of the transcript assembly, high-quality reads were mapped back to assembled transcripts. Results indicated that 92.28%–93.42% of the reads from each sampled time point could be mapped to the assembled transcripts, indicating that the majority of reads had been utilized in the assembly.

### Transcriptomic profiling of *P. hopeiensis*

**Figure 1:**
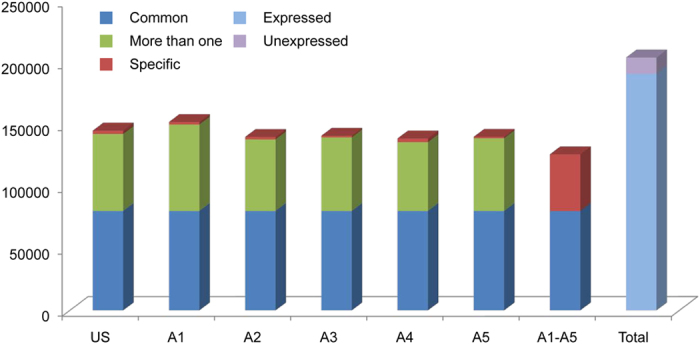
Summary of transcript profiles in *P. hopeiensis*. ‘Common’ represents transcripts expressed at all six time points, ‘More than one’ represents transcripts expressed at more than one time point but not all, ‘Specific’ represents transcripts expressed only only at the indicated time point, ‘Expressed’ and ‘Unexpressed’ represents transcript expression in the entire transcript assembly. US = Time 0 – prior to ABA treatment; A1-A5 = 1, 4, 8, 12, and 24 h after ABA treatment.

Transcript expression levels were estimated based on their FPKM values. The majority of the assembled transcripts (191,394, 93.64%) exhibited an expression level ≥0.01 in at least one time point (Fig. 1). This result was in a good agreement with the high utilization of high-quality reads. All six time points had a relatively high number of expressed transcripts, ranging from 138,987 (68.00%) to 152,343 (74.54%). The first time point (A1) after ABA treatment had the highest number of transcripts. In total, 80,217 transcripts were expressed in all six sampled time points.

ABA plays a pivotal role in response to osmotic stress, regulating a variety of downstream transcriptional regulatory networks^11^. Previous reports have demonstrated that exogenous application of ABA could induce a number of dehydration-responsive genes^10^,^17^. In the present study, only a small number (1,132–2,972; 0.59%-1.55%) of transcripts were uniquely expressed at a specific, single time point. Interestingly, only 2,803 (1.37%) transcripts were uniquely expressed at Time 0 (US) prior to ABA application, while 45,946 transcripts were uniquely expressed in the time points (A1-A5) after ABA treatment, representing 24.01% of the entire identified transcriptome. This indicates that many extremely low abundant transcripts characterized as unexpressed at Time 0 (US) were detected after ABA treatment, suggesting that they may play a central role in abiotic stress response. These results are consistent with a previous report in *Arabidopsis*^12^ which demonstrated that significant and dynamic transcriptomic changes occur in the trancriptome in response to ABA treatment.

### Validation of transcript abundance

**Figure 2:**
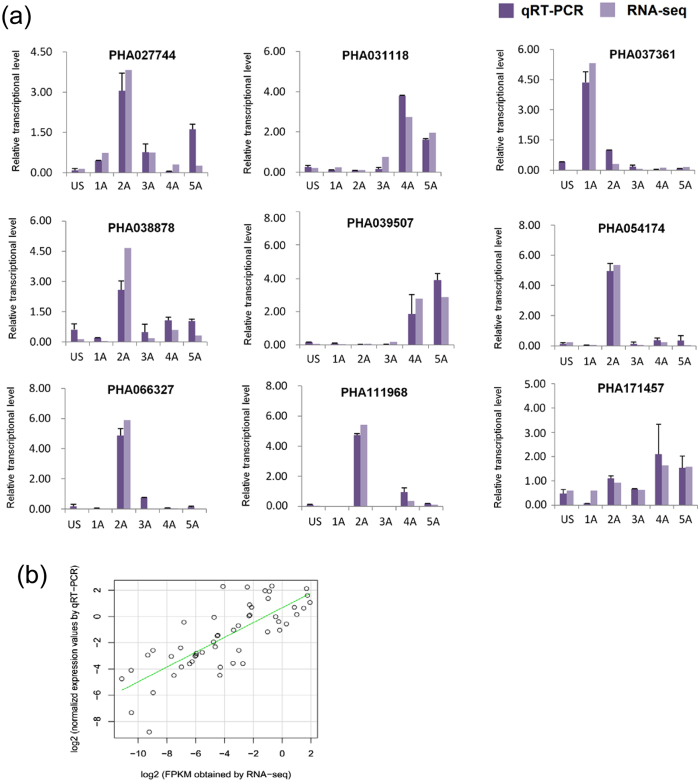
Comparison of the relative abundance of nine selected transcripts as determined by RT-qPCR and FPKM values for all six time points. (**a**) Relative transcript abundance of nine selected transcripts at all six time points as determined by RT-qPCR and RNA-seq. (**b**) Correlation between relative abundance values obtained by RT-qPCR and RNA-seq.

RT-qPCR was used to validate the level of transcript abundance obtained by RNA deep sequencing and the resulting FPKM values. Nine transcripts were selected for RT-qPCR analysis and their relative abundance was measured at all six time points (Fig. 2a). Results indicated that the relative levels of transcripts obtained by RT-qPCR were in a good agreement with values produced from the RNA-seq data. The correlation coefficient was 0.78 for the data obtained by the two approaches for estimating the expression levels of the nine selected transcripts at all six time points (Fig. 2b).

### Measurement of ABA levels

**Figure 3:**
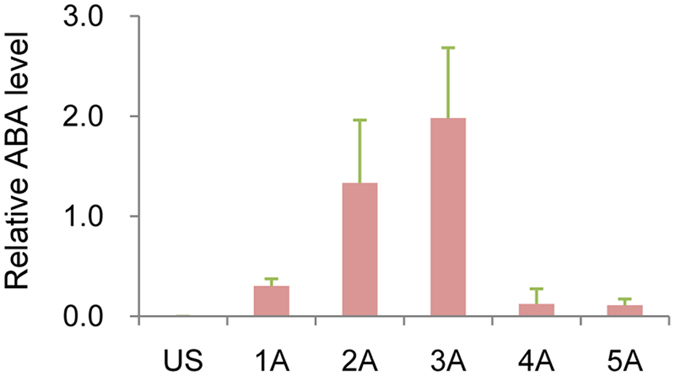
ABA concentration in leaves of *P. hopeiensis* at all six time points sampled in the present study. US = Time 0 – prior to ABA treatment; A1-A5 = 1, 4, 8, 12, and 24 h after ABA treatment.

The ABA concentration in the treated plants was also measured at all six time points (Fig. 3). ABA concentration in the plants was undetectable at Time 0 (US) and then increased, reaching a peak at 8 h (3 A) after treatment and then significantly decreasing at 12 and 24 h. This data indicates that the potential effect of ABA most likely occurred during the early time point following ABA treatment. Collectively, the data indicate that exogenous application of ABA induced changes in gene expression as evidenced by the composition of the transcriptome at different time points following ABA treatment. Interestingly, the expression trend of some transcripts was similar to the change of ABA content, such as PHA027744, PHA037361, PHA038878, PHA054174, PHA066327, and PHA111968 (Figs. 2 and 3). It indicates that these transcripts played roles in the response to ABA. For example, PHA037361 was a member of AREB/ABF family.

### Differentially expressed transcripts and functional annotation

R package ‘EdgeR’^18^ was used to identify the differentially expressed transcripts between two adjacent samples in chronological order. As expected, a large number of transcripts were identified as differentially expressed between untreated (Time 0) and ABA-treated samples (Figure S2). Results indicated that a relatively significant change (19.38–22.19%) in transcription occurred during the first 8 hours after ABA treatment. In contrast, only 8.95% of the transcripts were differentially expressed between 12 and 24 h (4 A and 5 A). The data indicate that there was a fairly rapid response to ABA but that a new equilibrium in gene expression was established as evidenced by fewer changes in gene expression during the latter time points of the study.

**Figure 4:**
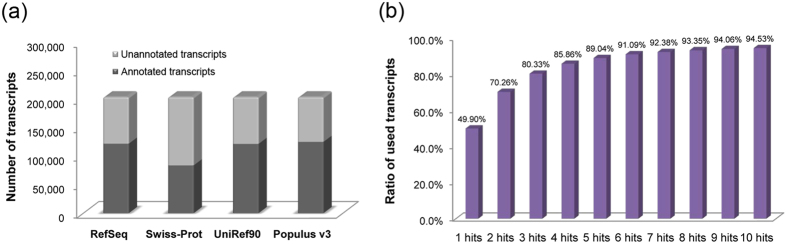
Global view of transcript annotation in *P. hopeiensis*. (**a**) Number of annotated transcripts identified in *P. hopeiensis* by blasting against four different databases. (**b**) Sequences similarity comparison between *P. trichocarpa* and *P. hopeiensis.* The labels on the x axis represent the number of hits in *Populus* v3.

To annotate the assembled transcripts, transcript sequences were blasted against four databases, including RefSeq^19^, Swiss-Prot^20^, UniRef90^21^, and *Populus* v3^1^, to identify homologous sequences. The number of homologues identified in Swiss-Prot was relatively low (Fig. 4a) with approximately 41.04% of the transcripts having at least one hit. This may be due to the fact that Swiss-Prot is fully composed of manually annotated entries^20^. In contrast, more than half of the assembled transcripts (59.57–61.30%) had at least one homologous sequence in the other three databases. The *Populus* v3 database contributed the highest number of homologous sequences (125,288 best hits), which is higher than the number of identified transcripts in *P. trichocarpa* (73,013)^1^. The best hits (sequences with the highest sequence similarity) in *Populus* v3 (*P. trichocarpa*) were analyzed and the results indicated that only 36,433 (49.90%) of the transcripts in *P. trichocarpa* were used to constitute the set of transcripts with the highest similarity to transcripts of *P. hopeiensis*. This suggests that a high degree of similarity exists between the *P. trichocarpa* and the assembled transcripts of *P. hopeiensis. P. trichocarpa* was the first tree species whose genome was sequenced and remains the only sequenced genome in the *Populus* genus^1^. For this reason, among others *P. trichocarpa* has served as a model species for the genomic studies of woody plants and maintains an important role^1^,^22^. To further analyze the homology between *P. trichocarpa* and *P. hopeiensis*, the alignment of transcripts from *P. hopeiensis* with *Populus* v3 was reanalyzed by altering the number of hits from 1 to 10 (Fig. 4b). Results indicated that the transcripts used in *Populus* v3 increased significantly, rising to almost 94.53%, as the number of hits rose from 1 to 10. These data indicated that a high degree of sequence similarity exists between *P. trichocarpa* and *P. hopeiensis*.

### Gene ontology enrichment and metabolic pathway analysis

**Figure 5:**
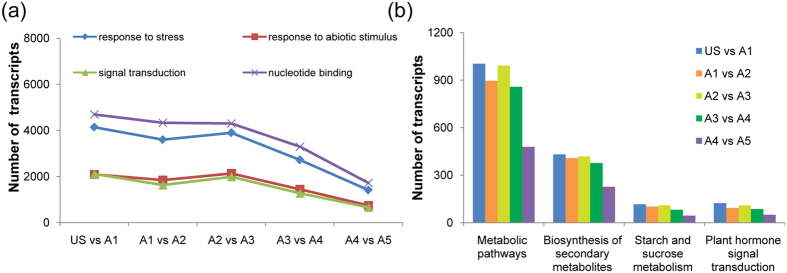
Dynamic changes in the assignment of transcripts of *P. hopeiensis* to GO enrichment categories and KEGG metabolic pathways in response to ABA treatment. (**a**) GO enrichment in four categories. (**b**) Dynamic changes in four major pathways in response to ABA treatment.. US = Time 0 – prior to ABA treatment; A1-A5 = 1, 4, 8, 12, and 24 h after ABA treatment.

Gene onotology^1^ annotations were performed to describe the functional characteristics of assembled data in *P. hopeiensis*. In this study, Swiss-Prot was selected for GO annotations of *P. hopeiensis* because of its stringent standards^20^. GO annotations of the best Blast hits of the assembled transcripts of *P. hopeiensis* in Swiss-Prot were assigned and then mapped into GO slim. Results indicated that GO slim entries based on all of the assembled transcripts (all six time points) were distributed in a variety of functional categories (Figure S3). To investigate changes in response to ABA treatment, GO enrichment results were compared between every two successive time points in chronological order. Results revealed that the number of transcripts in four enriched GO terms, response to stress, response to abiotic stimulus, signal transduction, and nucleotide binding, exhibited significant increases during the first 8 hours after ABA treatment which then significantly decreased at 12 and 24 h (Fig. 5a). It is possible that this resulted from the plants becoming insensitive to the ABA or limiting its uptake since ABA levels were also very low at 12 and 24 h.

KEGG analysis is a method that can be used to anchor transcripts to known metabolic pathways. Using the KEGG Automatic Annotation Server (KAAS), 319 pathways were detected in the whole transcriptome (all six time points) of *P. hopeiensis*. Most of the transcripts (16.66%) were anchored to two different categories (Figure S4), metabolic pathways and biosynthesis of secondary metabolites. To further analyze changes in the number of transcripts assigned to different KEGG categories in response to ABA treatment, KEGG results were compared between every two successive time points. The analysis demonstrated that both metabolic pathway and the biosynthesis of secondary metabolites categories were immediately enriched (US vs. A1) in response to the ABA treatment and maintained the high levels of enrichment during the duration of the study (Fig. 5b). These data indicate that there was a strong increase in metabolic activity in response to the ABA treatment. In addition to the significant changes in metabolic activity reflected by the KEGG analysis, changes in the starch and sucrose metabolism pathway during the first 12 hours after ABA treatment suggests that there was significant increase in energy consumption, which is a very important feature in the adaptation of plants to abiotic stress. The plant hormone signal transduction pathway was also enriched in response to the ABA treatment, especially in the first 12 hours, suggesting that this shift also played a significant role in the response to ABA treatment.

### Global view of transcription factor

**Figure 6:**
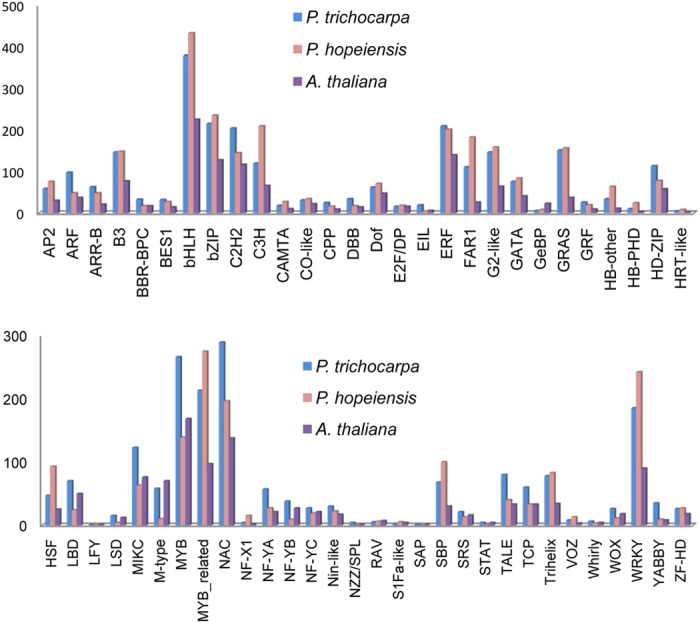
The number of transcription factors in *P. trichocarpa, P. hopeiensis* and *Arabidopisis thaliana* distributed in each of the TF families as defined by the PlantTFDB.

Transcription factors (TFs) play important roles in stress response by regulating the transcription of collections of specific target genes. TFs were identified from the assembled transcripts of *P. hopeiensis* using the PlantTFDB 3.0 pipeline^23^. In total, 4,037 TFs, belonging to 56 families were identified in the whole transcriptome (Fig. 6). *P. hopeiensis* and *P. trichocarpa* have similar numbers and types of TFs. Both species have greater numbers of TFs than reported for *A. thaliana*^23^ in the majority of TF families, implying that a more complex regulatory network exists in woody plants than in herbaceous plants.

**Figure 7:**
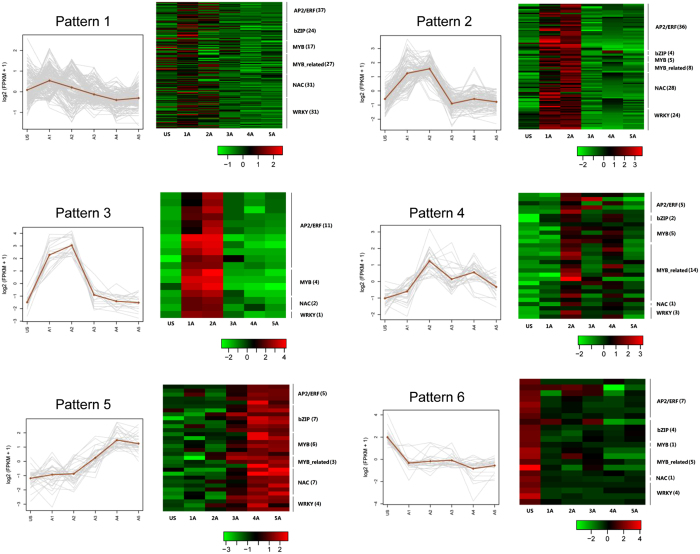
Different patterns of expression of TFs in *P. hopeiensis* in response to ABA treatment. The TFs represented in each pattern belong to a different TF gene family. US = Time 0 – prior to ABA treatment; A1-A5 = 1, 4, 8, 12, and 24 h after ABA treatment. The color scale at the bottom of each heat map represents the transformed FPKM value.

Many families of TFs, such as AP2/ERF^24^, NAC^25^, MYB, MYB_related^26^, bZIP^27^ and WRKY^9^, etc., play central roles in plant response to abiotic stress by regulating the expression of downstream genes. In this study, six TF patterns were observed based on the expression of the above six TFs families in *P. hopeiensis* in response to ABA treatment (Fig. 7). In total, 366 differentially expressed TFs were identified. The result showed that the majority of differentially expressed TFs increased rapidly (Pattern 1–3) after ABA treatment (Fig. 7). Most exhibited relatively moderate fold changes as shown in Pattern 1. TFs in Pattern 3 exhibited the greatest fold increase, as well as the greatest fluctuation. Pattern 3 was comprised of 18 TFs, and 11 of them were AP2/ERF family members. Pattern 4 was comprised of 30 TFs whose expression level peaked after 4 hours and then slowly decreased. Pattern 5 was comprised of 32 TFs that increased during the later time points of the study, therefore representing a slow adaptive response. Finally, pattern 6 was comprised of 22 TFs whose expression level were elevated prior to the ABA treatment (Time 0) and then slowly decreased during the time points following ABA treatment.

As mentioned above, many AP2/ERF superfamily members from various plant species have been reported to be involved in abiotic stress responses^24^. Among the 366 differentially expressed TFs in this study, 277 transcripts were identified as AP2/ERF TFs. Many AP2/ERF members responded rapidly to the ABA treatment and were highly expressed at the 1 and 4 h time points (A1-A2). This pattern was also observed for other abiotic-stress-related TF families, such as bZIP, NAC, and MYB. As a key component in ABA signaling in plants, WRKY family members act as both repressors and activators and thus play a role in the repression and de-repression of important plant processes^9^. One of the patterns observed in the current study indicated that the expression levels of 22 TFs decreased in response to ABA treatment. These TFs may act as repressors under non-stressed conditions and their down regulation in response to ABA may de-repress stress-related plant processes. Overall, the patterns of expression of TFs belonging to different TF families indicate their important role in plant response to ABA and perhaps the drought-tolerance trait exhibited by *P. hopeiensis*.

## Conclusions

This study describes the genome-wide transcriptional response of *P. hopeiensis* to the application of ABA. This work represents the genome-wide study to characterize the transcriptional responses of *P. hopeiensis* to the application of ABA. Since this work describes the global effects of ABA-induced changes in a tree species, and poplar in particular, this study will serve as a great resource for further NGS studies on the regulation of stress response in trees, and also provides data that will assist in whole genome assembly and annotation of *P. hopeiensis*. The present study will help to develop a better understanding of abiotic stress-related mechanisms and the broad functions of transcriptional regulatory networks in *P. hopeiensis*, other *Populus* species, and woody plants in general.

## Methods

### Plant materials

Tissue-cultured plants of *P. hopeiensis* were grown and synchronized (using vegetative stem segments containing an axillary bud) on 1/2 MS medium supplemented with 0.4 mg l^−1^ IBA in a growth chamber at 25 °C under LD conditions (16-h light/8-h dark photoperiod, cool white fluorescent light, 250 μmol m^−2^ s^−1^). After one month, plantlets of *P. hopeiensis* were selected for ABA treatments and were used as experimental material. The roots of samples were submerged in a 100 μM ABA solution^28^,^29^,^30^. The leaves of *P. hopeiensis* were harvested at 1, 4, 8, 12, and 24 h (A1-A5) and plants sampled prior to submergence in the ABA solution served as controls (Time 0 or US). Leaves from a single plant served as a biological replicate and three biological replicates were collected at each time point, including Time 0 (US).

### RNA preparation

All samples were immediately placed in liquid nitrogen and stored at −80 °C until RNA extraction. Total RNAs of each sample was extracted using a modified CTAB method^31^. The total RNA was then pretreated with RQ1 DNase (Promega, Madison, WI, USA) to remove genomic DNA contaminants.

### Deeping sequencing and preliminary processing of data

A Total RNA sample sequencing library of each stage time point was constructed using a TruSeq^®^ RNA sample preparation kit v2 (Illumina, San Diego, CA, USA) following the manufacturer’s instructions. Then 2 × 100 base-pair (bp) paired-end sequencing of each library was performed using an Illumina Hiseq 2000 platform in accordance with Illumina’s protocols provided by the manufacturer. Subsequently, NGS toolkit software (v2.3) was used^16^, employing default parameters, to filter out low-quality reads in the raw data. The remaining high-quality reads were then used for the assembly and downstream processing.

### Transcript assembly, estimation of abundance, and functional annotation

Trinity^32^ software (an assembly program based on the de Bruijn graph theory, was used for paired-end assembly with default or optimal parameters) was used for *de novo* assembly and construction of the whole transcriptome. High-quality reads from all six time points were combined as input data to improve the overall quality of the assembled transcriptome, instead of assembling each time point individually. After assessing different *k*-mer sizes, 25-mer yielded the best assembly for the desired application. Assembled transcripts ≥200 nt were used for the calculation of abundance and functional annotation.

The fragments per kilobase of transcript per million mapped reads (FPKM) statistical method was used to estimate transcript abundance and to identify differentially expressed transcripts at each successive time point in the study. The identification of differentially expressed transcripts was carried out using the edgeR method^18^ and the resulting p-values were adjusted using the False Discovery Rate (FDR) method at a significance level of 0.05. To annotate the transcripts, the sequences of the assembled transcripts were blasted against four different databases, RefSeq^19^, Swiss-Prot^20^, UniRef90^21^ and *Populus* v3^1^. An E-value of 1E-5 was adopted as the threshold^33^.

### RT-qPCR verification

To validate transcript abundance as reflected by FPKM values for the assembled transcripts, nine transcripts were selected and subjected to RT-qPCR analysis to determine estimates of relative expression. RT-qPCR was performed with an ABI PRISM 7500 Fast Real-time PCR System (Applied Biosystems, Foster City, CA, USA) using a SYBR Premix Ex TaqTM Kit (TaKaRa, Kyoto, Japan) with amplification conditions as recommended by TaKaRa. All reactions were run in triplicate for each sample, and the *Populus* ACTIN (Accession: AY261523.1) was used as a reference gene for normalization^34^. All primers used in the RT-qPCR analysis are listed in Table S1.

### Measurement of ABA

For each time points, the sample of three replicates from leaves was measured for ABA content. [^2^H_6_] ABA was used as an internal standard for ABA. Leaves were frozen in liquid nitrogen and freeze-dried. Samples were homogenized in 80% (v/v) methanol and kept at 4 °C overnight. After filtration through a Whatman No. 1 filter paper, the extracts were concentrated to less than 1 ml under vacuum at 35 °C. The concentrate was then taken up in 3 × 3 ml of 0.4% (v/v) acetic acid in distilled water and injected into a Sep-Pak C_18_ cartridge. Plant hormones were eluted from the Sep-Pak with 70% (v/v) methanol in 0.4% (v/v) acetic acid in distilled water. The eluate was then dried under vacuum. The dry residue was dissolved in 2 ml of 20% (v/v) methanol in 0.4% (v/v) acetic acid in distilled water. The ABA content was then analyzed by high-performance liquid chromatography (HPLC), using an Agilent 1110 Series HPLC (Agilent Technologies, Santa Clara, CA, USA), Waters C_18_ column, methanol-water (70:30), pH 4.0 as mobile phase and a flow rate of 0.5 ml min^−1^. Each sample was assayed using three replicates.

### Gene ontology enrichment analysis and KEGG pathway assignment

Annotations obtained by blasting against the Swiss-Prot database were used for gene ontology^1^ assignment and plant GO slim as defined by the GO Consortium^35^. The KEGG Automatic Annotation Server (KAAS) was used to analyze the annotated transcripts and place them into presently-known pathways^36^. This was performed for the entire transcriptome (all six time points combined). Differentially expressed transcripts were subjected to GO enrichment analysis and pathway. The hypergeometric test with FDR adjustment was used to identify significantly different enrichment sets with significance level of 0.05.

### Transcription factor identification

Transcription factors (TFs) are grouped into families based on their conserved DNA-binding domains (DBDs). Based on these signature domains, plant TFs are classified into 58 families in PlantTFDB^23^. Two steps were used to identify TFs from the assembled transcripts. First, the ESTScan^37^ was used to predict protein sequences from each of the assembled transcripts. Secondly, the predicted protein sequences were used in the TF identification pipeline of PlantTFDB 3.0 and the resulting TFs were assigned into families according to PlantTFDB family assignment rules^23^. Patterns of TF expression were constructed based on the transformed FPKM (log2 (FPKM + 1)) values. EdgeR Bioconductor package in R was used for generating hierarchical clustering and heatmaps.

## Additional Information

**How to cite this article**: Chen, Z. *et al*. Dynamic changes in the transcriptome of *Populus hopeiensis* in response to abscisic acid. *Sci. Rep.* **7**, 42708; doi: 10.1038/srep42708 (2017).

**Publisher's note:** Springer Nature remains neutral with regard to jurisdictional claims in published maps and institutional affiliations.

## Supplementary Material

Supplementary Information

## Figures and Tables

**Table 1:** Summary of RNA sequencing data.

Sample	Raw reads	High-quality reads	Utilized reads for assembly
Unstressed (US)	38,619,524	34,751,596 (89.98%)	32,131,623 (92.46%)
1 h (A1)	44,748,632	39,582,354 (90.13%)	36,656,421 (92.61%)
4 h (A2)	40,970,338	36,685,730 (89.17%)	33,853,915 (92.28%)
8 h (A3)	37,701,922	33,678,146 (87.76%)	31,330,127 (93.03%)
12 h (A4)	38,598,518	34,305,030 (88.84%)	31,891,353 (92.96%)
24 h (A5)	44,364,118	39,604,004 (88.87%)	36,997,495 (93.42%)
Total	245,003,052	218,606,860 (89.23%)	202,860,934 (92.80%)
