# Supplementary material for: Dynamic changes in the transcriptome of Populus hopeiensis in response to abscisic acid
Source: Sci Rep. 2017 Feb 15;7:42708. doi: 10.1038/srep42708 (PMC5309881; doi:10.1038/srep42708)
Supplement: Supplementary Information [file srep42708-s1.pdf]

Supplementary Information:

Dynamic changes in the transcriptome of *Populus hopeiensis* in response to abscisic acid

Zhong Chen[1†], Lexiang Ji[1,2†], Jia Wang[1†], Jinpu Jin[3], Xiaoyu Yang[1], Pian Rao[1], Kai Gao[1], Weihua Liao[1], Meixia Ye[1], Xinmin An[1]*

[1]National Engineering Laboratory for Tree Breeding, Key Laboratory of Genetics and Breeding in Forest Trees and Ornamental Plants of the Ministry of Education, The Tree and Ornamental Plant Breeding and Biotechnology Laboratory, College of Biological Sciences and Biotechnology, Beijing Forestry University, Beijing, 100083, P. R. China

[2]Institute of Bioinformatics, University of Georgia, Athens, GA 30602, USA

[3]State Key Laboratory of Protein and Plant Gene Research, Center for Bioinformatics, College of Life Sciences, Peking University, Beijing, 100871, P. R. China

[†]These authors contributed equally to this work.

* To whom correspondence should be addressed.

Tel/Fax: +86-010-62336248;

E-mail: anxinmin@bjfu.edu.cn

Supplementary Information

Figure S1 Distribution of assembled transcript lengths in *Populus hopeiensis*.

Figure S2 Differentially expressed transcripts in *Populus hopeiensis* in response to ABA.

Figure S3 Gene ontology distribution for the assembled transcripts in *Populus hopeiensis*.

Figure S4 Distribution of the assembled transcripts of *Populus hopeiensis* in the top ten metabolic pathways as determined by the number of transcripts placed in each enrichment category.

Table S1 Primers used for RT-qPCR analysis.

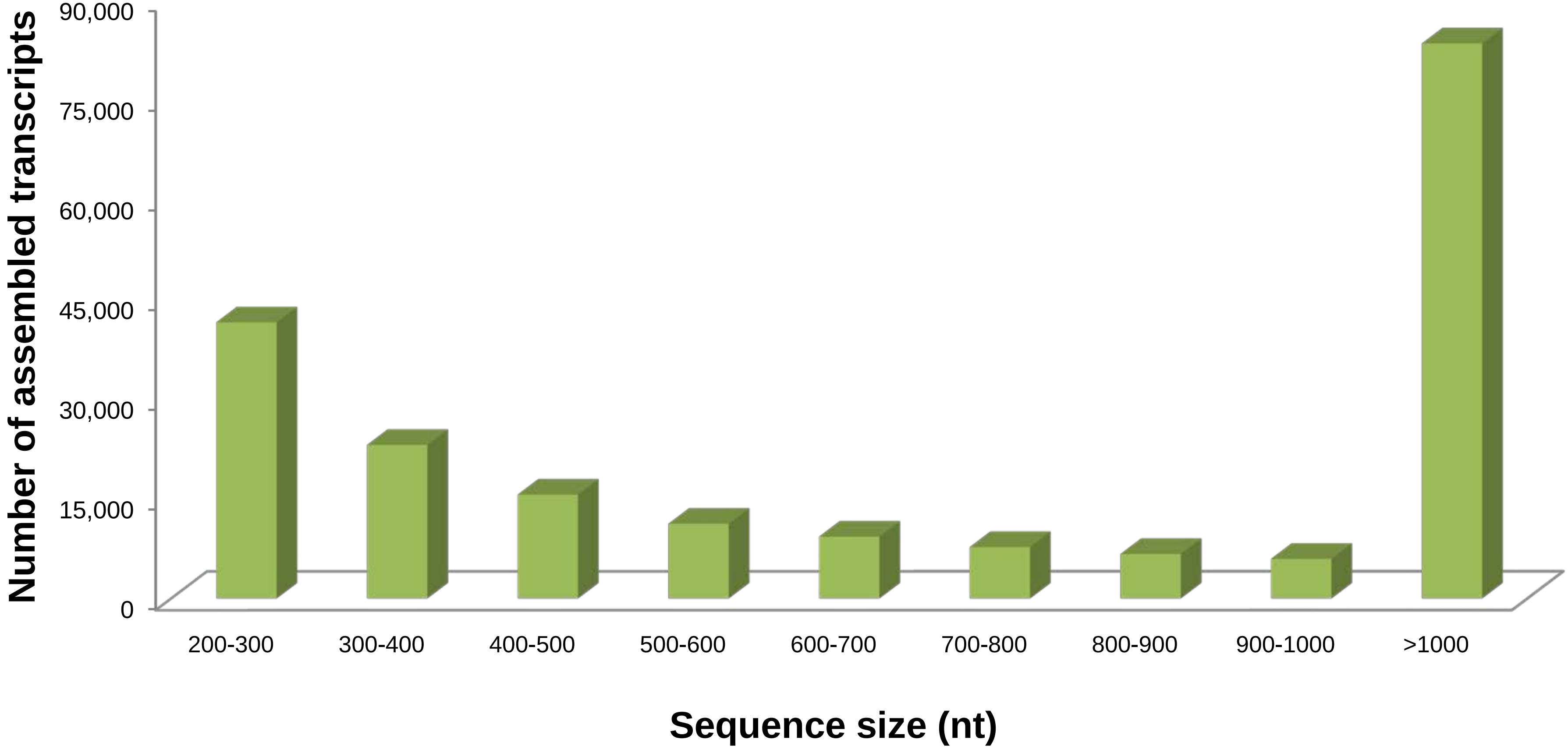

Figure S1 Distribution of assembled transcript lengths in *Populus hopeiensis*

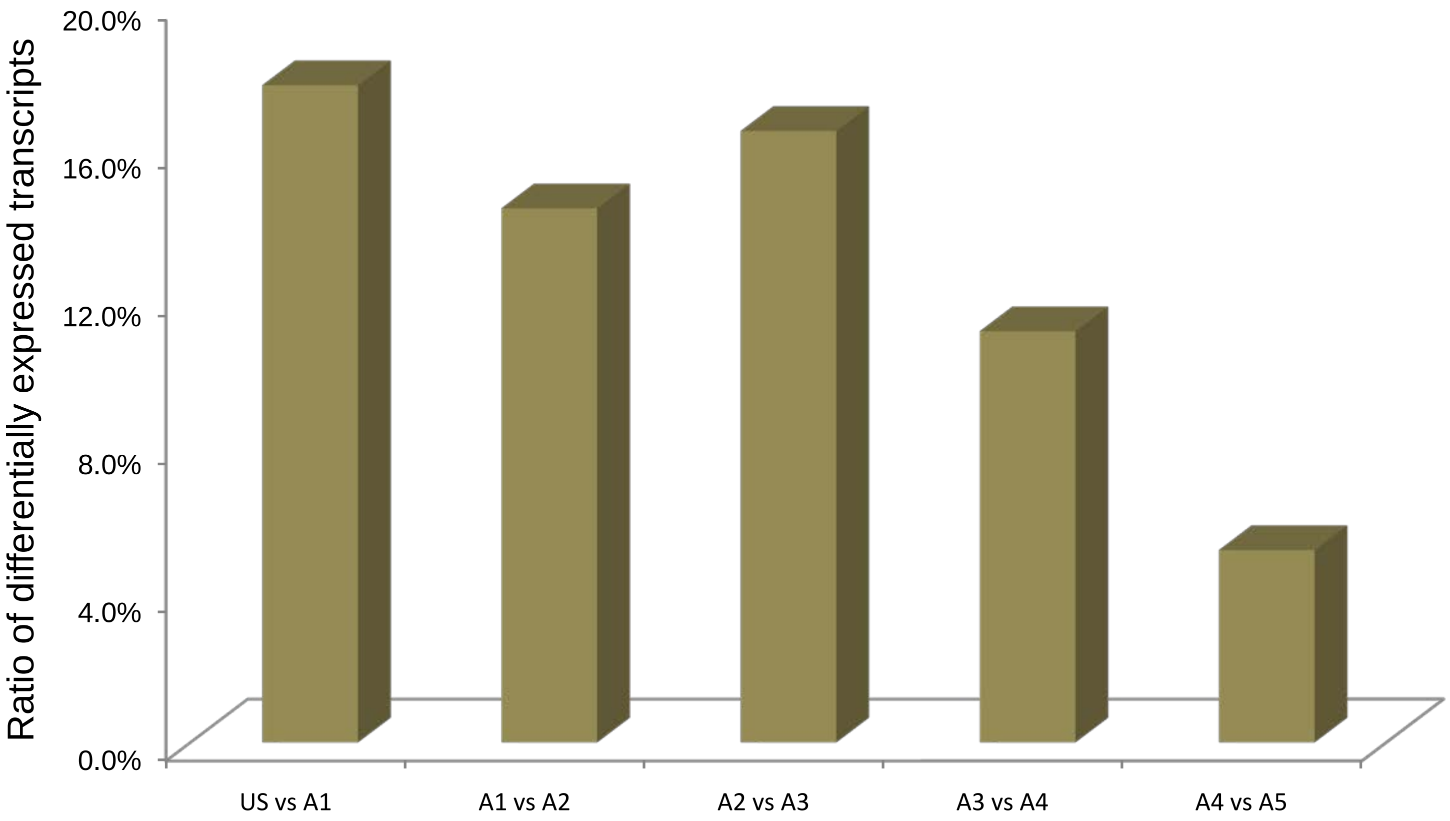

Figure S2 Differentially expressed transcripts in *Populus hopeiensis* in response to ABA.

Each bar represents differences in transcript expression between one time point and the successive time point. Six time points were evaluated, Time 0 (US) prior to ABA application, 1, 4, 8, 12, and 24 h (A1 – A5, respectively).

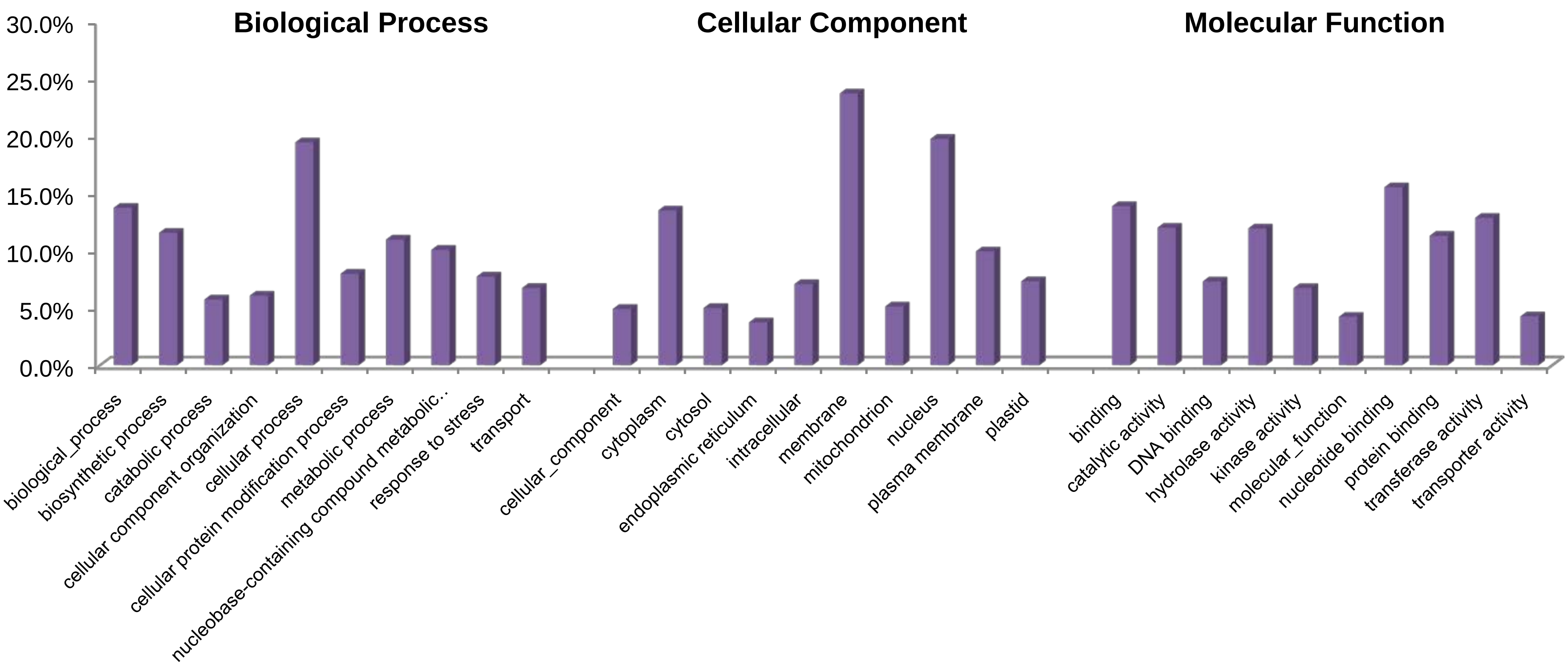

Figure S3 Gene ontology distribution for the assembled transcripts in *Populus hopeiensis*.

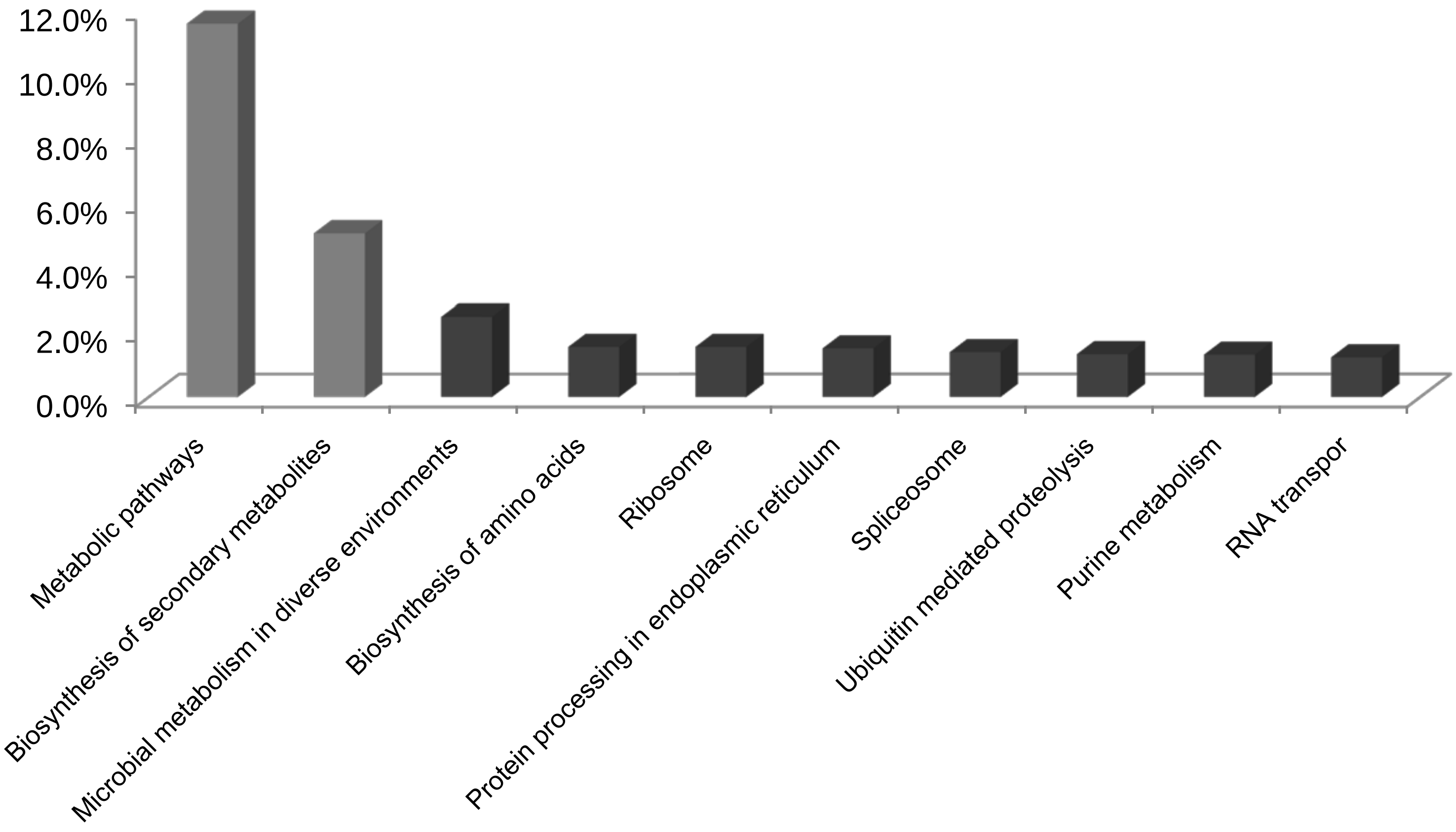

Figure S4 Distribution of the assembled transcripts of *Populus hopeiensis* in the top ten metabolic pathways as determined by the number of transcripts placed in each enrichment category

Table S1 Primers used for RT-qPCR analysis

Name	Forward (5'-3')	Reverse (5'-3')
PHA027744	TCCTCATCTCACGCAAACAG	TGGGGTTTTGAAGATGGTGG
PHA031118	TGCTTGAAATGTCCTCCCAC	GCTGGTCAAGACTTACGAGG
PHA037361	GTGCCCTTCTCATGTGGAAG	TACTTCCTCGAGCAAAGTCG
PHA038878	GCAAAGAACAGGAGGGGAAA	CTTGACCTGGGCCTTCTTTT
PHA039507	TGTAAAGCAACATGGGAGCA	CACTCGAAGGCCTGTTTTCT
PHA054174	CACTCATGCCTCACTCTCAC	TGCCAGGAAATGCAAAGTCT
PHA066327	TCCTTGGTTTGAGCCCTTTC	GTCACTCCTCTTAGCGTTGG
PHA111968	CTTCTCAGTCCTGCTCGTTC	GAGAGAGTGCCACATGATCG
PHA171457	GAGGCTCACGTTTTCAAAGC	TGCTCCTCTCAATTCGATGC
Populus ACTIN	CTCCATCATGAAATGCGATG	TTGGGGCTAGTGCTGAGATT
